# The Integral Role of Genetic Variation in the Evolution of Outcrossing in the *Caenorhabditis elegans-Serratia marcescens* Host-Parasite System

**DOI:** 10.1371/journal.pone.0154463

**Published:** 2016-04-27

**Authors:** Raymond C. Parrish, McKenna J. Penley, Levi T. Morran

**Affiliations:** 1 Department of Biology, Indiana University, Bloomington, Indiana, United States of America; 2 Department of Biology, Emory University, Atlanta, Georgia, United States of America; Fred Hutchinson Cancer Research Center, UNITED STATES

## Abstract

Outcrossing is predicted to facilitate more rapid adaptation than self-fertilization as a result of genetic exchange between genetically variable individuals. Such genetic exchange may increase the efficacy of selection by breaking down Hill-Robertson interference, as well as promoting the maintenance of within-lineage genetic diversity. Experimental studies have demonstrated the selective advantage of outcrossing in novel environments. Here, we assess the specific role of genetic variation in the evolution of outcrossing. We experimentally evolved genetically variable and inbred populations of mixed mating (outcrossing and self-fertilizing) *Caenorhabditis elegans* nematodes under novel ecological conditions—specifically the presence of the virulent parasite *Serratia marcescens*. Outcrossing rates increased in genetically variable host populations evolved in the presence of the parasite, whereas parasite exposure in inbred populations resulted in reduced rates of host outcrossing. The host populations with genetic variation also exhibited increased fitness in the presence of the parasite over eight generations, whereas inbred populations did not. This increase in fitness was primarily the result of adaptation to the parasite, rather than recovery from initial inbreeding depression. Therefore, the benefits of outcrossing were only manifested in the presence of genetic variation, and outcrossing was favored over self-fertilization as a result. As predicted, the benefits of outcrossing under novel ecological conditions are a product of genetic exchange between genetically diverse lineages.

## Introduction

Within the tree of life, the abundance of species that reproduce via outcrossing is surprising due to the inherent cost of outcrossing relative to self-fertilization or asexual reproduction [1–6]. Outcrossing individuals generally incur either the cost of males [3, 6] or the cost of meiosis [2, 6, 7], which are both substantial, and these costs must be overcome for outcrossing to be maintained in a population. Therefore, outcrossed offspring must be significantly more fit than offspring produced by selfing or asexual reproduction [2, 3, 8]. Patterns consistent with the benefits of outcrossing outweighing these inherent costs have been observed when organisms are under selective pressure to rapidly adapt in the field [9–13] as well as in the laboratory [14–19]. Furthermore, experimental studies have demonstrated that exposure to novel environments can also select for outcrossing over self-fertilization [20–23].

The advantage of outcrossing, relative to self-fertilization, in a novel environment may be due to the contrasting population genetic consequences of outcrossing versus self-fertilization. [3, 8, 24, 25]. This potential population genetic advantage of outcrossing lies in the fact that outcrossing facilitates genetic exchange between lineages whereas self-fertilization does not allow for such exchange. In particular, outcrossing presents the opportunity to break down Hill-Robertson interference (reviewed in [26]), whereas selfing should perpetuate the interference by maintaining linkage disequilibrium. Outcrossing can increase the efficacy of selection by breaking down linkage between beneficial and deleterious alleles while also potentially uniting beneficial alleles that originate in different lineages, thereby facilitating more rapid adaptation [27–32]. Additionally, outcrossing may alleviate fitness loss due to inbreeding depression by incorporating novel alleles into a lineage, whereas selfing can perpetuate homozygosity and the fitness costs of fixing homozygous deleterious alleles within a lineage [29, 31, 33]. Nonetheless prolonged periods of selfing may also reduce the degree of inbreeding depression over time by efficiently purging the mutation load [34–36]. Importantly, the genetic benefits of outcrossing rely on the presence of additive genetic variation, therefore genetic variation should play an integral role in outcrossing’s selective advantage over self-fertilization under novel conditions.

Multiple studies have demonstrated a selective advantage for outcrossing relative to self-fertilization under novel environmental conditions [37, 38]. Greater outcrossing rates evolved in mixed mating (self-fertilizing and outcrossing) *Caenorhabditis elegans* populations exposed to a novel, highly virulent strain of the bacterial parasite *Serratia marcescens*. Further, populations that outcrossed at elevated rates also exhibited significantly greater levels of adaptation to the parasite than populations that relied on self-fertilization [20]. Exposing *C*. *elegans* to coevolving *S*. *marcescens*, which may generate a consistently changing environment as hosts and parasites reciprocally adapt to one another, selected for the evolution and maintenance of greater outcrossing in *C*. *elegans* populations [21]. Additionally, obligate outcrossing conferred greater fitness in the presence of coevolving parasites than did mixed mating [39]. Similar empirical work, which utilized *C*. *elegans* and the bacterial parasite *Bacillus thuringiensis*, also found that outcrossing was maintained in host populations coevolving with parasites [22]. This trend was observed despite the fact that males, which facilitated outcrossing, were infected by the parasite more frequently and suffered greater mortality rates than hermaphrodites. However, outcrossing facilitated adaptation, therefore males and outcrossing were maintained despite the sex-specific effects of the parasite and the inherent costs of outcrossing.

Interestingly, *C*. *elegans* hosts did not exhibit significant levels of adaptation to *B*. *thuringiensis* parasites in one host-parasite coevolution study [40]. However, in this case the host populations started with limited standing genetic variation relative to many other *C*. *elegans* experimental coevolution studies. This lack of standing genetic variation has been shown to be disadvantageous for outcrossing populations in other species. Specifically, inbred subpopulations of the fish, *Poeciliopsis monacha*, experienced significantly elevated rates of parasitic infection relative to outbreeding subpopulations, despite the fact that both subpopulations reproduced via outcrossing [41]. Thus, the benefits of outcrossing can outweigh the inherent costs during exposure to parasites, but importantly, this selective advantage appears to be dependent on genetic variation.

Our goal was to determine the role of genetic variation in conferring the benefits of outcrossing relative to self-fertilization in a novel environment, and thereby assess how the population genetic consequences of outcrossing contribute to the selective advantage of outcrossing over self-fertilization. Here, we tested the effects of both natural and ethyl methanesulfonate (EMS) induced standing genetic variation on rates of outcrossing and adaptation in *C*. *elegans* populations exposed to *S*. *marcescens*. Populations of nematodes harboring genetic variation and highly inbred populations were experimentally evolved in the presence of virulent *S*. *marcescens*. Outcrossing rates were measured and compared between populations with initial genetic variation and the inbred populations. Then, we assessed the initial degree of inbreeding depression within populations and changes in fitness over the course of the experiment to determine if initial variation and the evolution of increased rates of outcrossing resulted in adaptation to the parasite.

## Materials and Methods

### Development of lines with and without variation

We acquired *Caenorhabditis elegans* strain EEV-A_0_ from the laboratory of Henrique Teotónio (Institut de Biologie de l’École Normale Supérieure, Paris, France). This strain, which contains genetic variation that has accumulated in natural populations of *C*. *elegans*, is the product of a funnel cross of sixteen different *C*. *elegans* strains that had previously been isolated from a wide range of locations around the world [23]. While all of the alleles found in the EEV-A_0_ strain do occur in strains isolated from natural habitats, it is important to note that many of the allelic combinations present in EEV-A_0_ do not occur in natural populations themselves. Nonetheless, highly deleterious allelic combinations were removed by natural selection during the construction of the strain [23]. The EEV-A_0_ strain will herein be referred to as “N” to signify that it contains allelic variation that is present in nature. After N was obtained, two subsets of this strain were inbred over eight generations to generate two isogenic lines (I1 and I2). Single L4 hermaphrodite nematodes were randomly chosen from each line and transferred each generation to serve as the sole parent to the following generation [42]. A subset of each of these inbred lines were then independently exposed to 10mM EMS over three successive generations as described in [20, 21] to infuse genetic variation into each population. EMS mutagenesis generated a mutation rate of approximately 2 x 10^−7^ per site per haploid genome each generation [43]. The mutagenized line derived from I1 was then labeled “M1” to signify that it contains chemically-induced standing genetic variation and is derived from the independently inbred group I1 nematodes, and the mutagenized line derived from I2 was labeled “M2” to signify that it contains chemically-induced standing genetic variation and is derived from the independently inbred group I2 nematodes.

### Experimental evolution

Each of these lines; N, I1, I2, M1, and M2; were then split into ten replicate populations of 1000 nematodes each. Five of the replicate populations from each line were passaged over eight nematode generations on *Serratia* Selection Plates [20] containing non-virulent, heat-killed Sm2170 *S*. *marcescens*. The other five replicate populations from each line were passaged over eight nematode generations on *Serratia* Selection Plates containing live, virulent Sm2170 *S*. *marcescens*. Only the offspring of nematodes that survived exposure to *S*. *marcescens* (either live or heat-killed, depending on the treatment) and reproduced were passaged to the next generation. Approximately 1000 L4 nematodes were transferred per replicate population each passage. The experimental evolution of all populations on both live and heat-killed Sm2170 treatments was performed concurrently under identical laboratory conditions wherein all populations were stored at 20°C for the duration of the experiment. A subset of each ancestral population was frozen and stored at -80°C prior to first round of selection. A subset of each experimental population was frozen and stored at -80°C after eight generations of selection. Experimental populations were started with 1000 individuals at approximately 5% male frequency. Initial male frequency was calculated by determining the male frequency of a liquid aliquot of each line in M9 buffer. The aliquot amount was scaled up to ensure transfer of 1000 individuals to each starting replicate population.

The experiment was executed for eight nematode generations because our goal was to test the effect of initial standing genetic variation on the evolution of outcrossing rates. Therefore, we wanted to assess the evolutionary dynamics within a short time frame to limit the number of *de novo* mutations in our populations.

### Outcrossing rates

The frequency of males in each population of *C*. *elegans* was recorded every four generations and subsequently converted to an outcrossing rate, correcting for the rate of spontaneous male production via X-chromosome nondisjunction, as described in Stewart and Phillips [44]. Outcrossing rate data violated the ANOVA assumptions of normality and equal variances. Therefore, we used the nonparametric sum-rank Wilcoxon test in JMP 10 to evaluate the effects of parasite treatment (live or heat-killed), nematode line (N, I1, I2, M1, and M2), and the interaction of treatment and line on outcrossing rates.

### Assessment of initial inbreeding depression

Assays were conducted to detect any initial inbreeding depression present in our ancestral populations that contained genetic variation (N, M1, and M2). First we either selfed five hermaphrodites per replicate population or mated five sperm-depleted hermaphrodites with 15 males per replicate population. L4 offspring that were either the product of self-fertilization (inbred) or cross-fertilization (outbred) were randomly chosen for fitness assays. Fitness assays were conducted by plating one hundred hermaphrodite nematodes of a single offspring type (either inbred or outbred) on *Serratia* Selection Plates [20] with 100 hermaphrodite nematodes of the GFP-expressing tester strain JK2735. After four days, the ratio of experimental line offspring to tester strain offspring was calculated for each plate, based on the frequency of individuals expressing GFP. These assays, designed to assess the relative fitness of inbred and outbred offspring of each line [20], were conducted in quadruplicate on both live, virulent *S*. *marcescens* and heat-killed, non-virulent *S*. *marcescens*.

We performed an ANOVA in JMP 10 testing the effects of parasite treatment (live or heat-killed), mating type (inbred or outbred), nematode line (N, M1, and M2), and all possible interactions of the main effects on the mean ratio of experimental to tester strain nematodes. We then performed linear contrasts testing the interaction of mating and line to detect the presence of inbreeding depression in each specific line.

### Assessment of evolved fitness

Fitness was assessed using competitive fitness assays, competing experimental populations against individuals from the GFP-labeled tester strain (JK2735) on the SSPs. The procedures followed those described in Morran et al (2009). Ancestral and experimental populations were thawed and allowed 2 generations of recovery time on their normal *Escherichia coli* (OP50 strain) food source. Then, nematodes from ancestral and experimental populations after eight generations of selection were competed against the tester strain. 100 individuals from an experimental population (or ancestral population) were mixed with 100 individuals of the tester strain. Competitive fitness was assessed by measuring the change in frequency of the GFP marker over the course of a single generation on the SSP containing live *S*. *marcescens*. Replicate populations were assayed at least two times. However, M1 replicate population # 2 from the live parasite treatment and M2 replication population # 4 from the live parasite treatment did not recover from freezing in sufficient numbers to permit a competitive fitness assay. Therefore, those replicate populations were not included in the analysis.

We present the change in mean fitness over eight generations exhibited by the competitive fitness of the experimental populations relative to the ancestral fitness. Each mean value represents the difference between the mean ratio of experimental offspring to tester strain offspring and the mean ratio of ancestral offspring to tester strain offspring, divided by the mean ratio of ancestral offspring to tester strain offspring. Therefore, all changes in mean competitive fitness are presented relative to the competitive fitness of the ancestral population. The ratios of experimental and ancestral offspring to tester strain offspring are greater in the experimental and ancestral inbred nematode lines (I1 and I2) due to reduced numbers of tester strain offspring. These lines were assayed at a later date than the other nematode lines. Different lab conditions or genetic changes in the tester strain could explain the reduced competitive fitness of the tester strain in these assays.

We performed an ANOVA in JMP Pro 10 (SAS Institute, Cary, NC) testing the effects of parasite treatment (live or heat-killed), nematode line, and the interaction between parasite treatment and nematode line on the percent change in mean fitness relative to the ancestor. Both parasite treatment and nematode line were treated as fixed effects. We then performed linear contrast tests to analyze the interaction of treatment and line to detect adaptation within each line.

## Results

### Outcrossing rates

**Fig 1:**
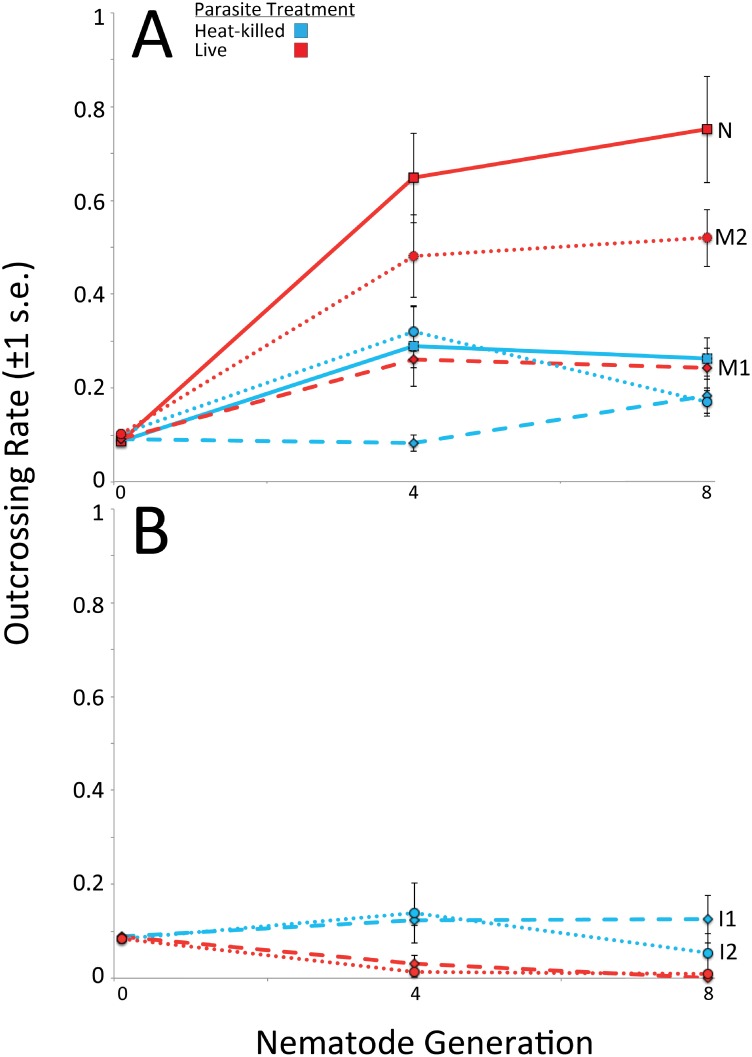
Elevated outcrossing rates in populations harboring variation. Outcrossing rates over generations of nematode evolution for populations of nematodes with (A) natural genetic variation (N) and mutagenesis-induced variation (M1 and M2) and (B) no initial variation (I1 and I2) when exposed to either live or heat-killed *S*. *marcescens*. Outcrossing rates were elevated in lines N, M1, and M2 when exposed to live parasites relative to exposure to heat-killed parasites. Conversely, outcrossing rates were observed to be lower in lines I1 and I2 when exposed to live parasites relative to exposure to heat-killed parasites.

To elucidate the role of initial genetic variation in the evolution of outcrossing, inbred nematodes (I1 and I2), nematodes with natural genetic variation (N), and nematodes with mutagen-induced genetic variation (M1 and M2), were experimentally evolved against *S*. *marcescens* for eight generations. The replicate populations harboring variation evolved elevated outcrossing rates after eight generations when exposed to live *S*. *marcescens* relative to when exposed to the heat-killed control (Fig 1A; M1 χ^2^_1_ = 3.94, P = 0.047; M2 χ^2^_1_ = 6.82, P = 0.009; N χ^2^_1_ = 6.82, P = 0.009). Conversely, the inbred lines did not evolve greater outcrossing rates in response to the live parasite (Fig 1B). Rather, I1 exhibited significantly lower outcrossing rates when exposed to the live parasite versus exposure to the heat-killed control after four (Fig 1B; χ^2^_1_ = 5.81, P = 0.016) and eight generations (Fig 1B; χ^2^_1_ = 7.26, P = 0.007). Further, I2 exhibited significantly lower outcrossing rates when exposed to the live parasite after four generations (Fig 1B; χ^2^_1_ = 4.04, P = 0.044) and no difference between parasite treatments after eight generations (Fig 1B; χ^2^_1_ = 3.36, P = 0.067). This reversal of parasite treatment effect on outcrossing rates indicates that genetic variation was integral to the evolution of elevated rates of outcrossing. Specifically, outcrossing was favored by selection in populations that harbored initial variation but was disadvantageous in the absence of variation.

### Assessment of initial inbreeding depression

**Fig 2:**
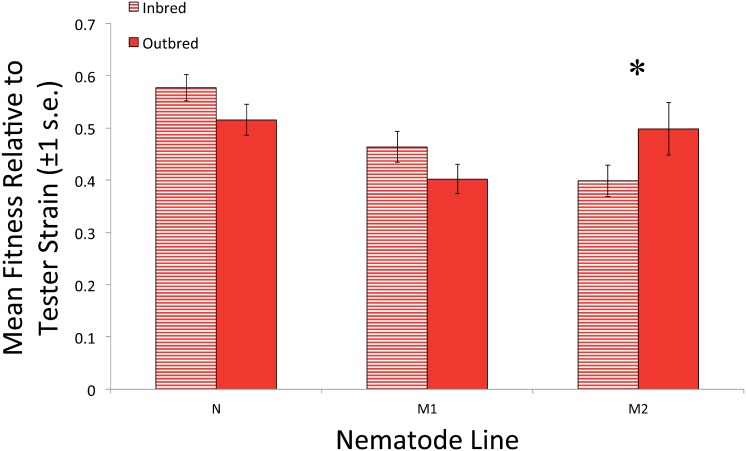
Initial inbred and outbred fitness in populations harboring variation. Percent mean fitness relative to the tester strain of offspring that are the product of self-fertilization (inbred) or outcrossing (outbred) from ancestral nematodes of all lines with initial genetic variation (N, M1, and M2). Mean fitness values represent the average fitness for each line in the presence of live and heat-killed parasites. The mean fitness of inbred offspring from N and M1 was equal or higher relative to outbred offspring, therefore these lines did not exhibit any detectable level of inbreeding depression. However, the mean fitness of inbred offspring from the M2 line was significantly lower than outbred offspring fitness, indicating that inbreeding depression was present in this line at the beginning of the experiment. *indicates a significant difference between mean values of inbred and outbred offspring in a given line.

The elevated rates of outcrossing within the populations harboring variation could be due to inbreeding depression. Outcrossing could be favored over selfing if self-fertilization results in significant inbreeding depression. Initial inbreeding depression was assessed in populations harboring variation via competitive fitness assays. The fitness of inbred and outbred offspring from ancestral nematodes of the N, M1, and M2 lines were compared in the presence of both live and heat-killed parasites. Overall, the fitness of inbred and outbred offspring was not significantly different in lines N (Fig 2 and Table 1; F_1,35_ = 2.30, P = 0.139) and M1 (Fig 2 and Table 1; F_1,35_ = 2.35, P = 0.134), regardless of the parasite treatment, thus indicating no significant levels of inbreeding depression. However, the fitness of inbred M2 offspring was significantly lower than the fitness of outbred M2 (Fig 2 and Table 1; F_1,35_ = 5.68, P = 0.023), regardless of the parasite treatment. Therefore, inbreeding depression was present in the M2 line.

**Table 1:** ANOVA table for inbreeding depression assay.

Source	Sum of Squares	df	Mean Square	F	P
Model	0.277	11	0.025	3.89	< 0.001
Error	0.227	35	0.006		
Total	0.504	46			
Treatment	0.034	1	0.034	5.30	0.03
Line	0.121	2	0.061	9.32	< 0.001
Mating Type	0.006	1	0.006	0.10	0.749
Treat X Line	0.031	2	0.016	2.39	0.107
Treat X Mating	0.001	1	0.001	0.18	0.675
Line X Mating	0.066	2	0.033	5.07	0.012
Treat X Line X Mating	0.027	2	0.014	2.11	0.137

### Assessment of evolved fitness

**Fig 3:**
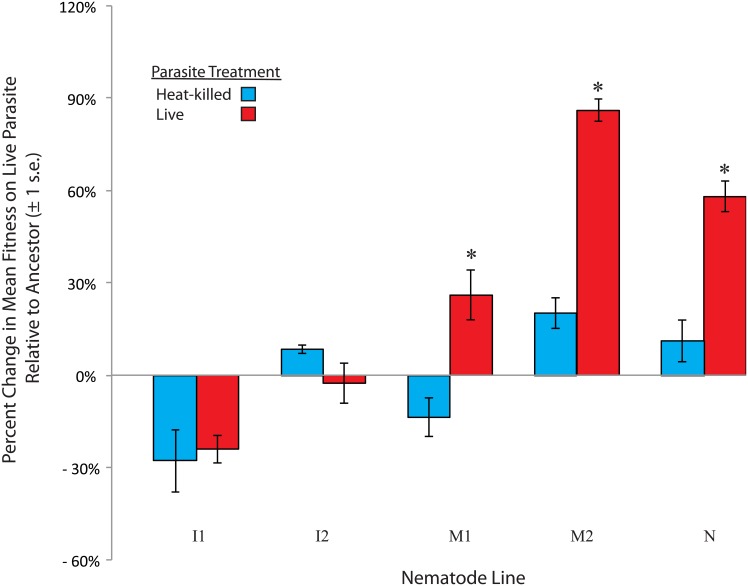
Adaptation in populations harboring variation. The mean change in competitive fitness in the presence of live *S*. marcescens, relative to ancestral fitness, was measured for each line after eight generations of selection. Lines that harbored initial variation (M1, M2, and N) and were passaged in the live parasite treatment exhibited significant increases in fitness relative to heat-killed controls. However, inbred host populations (I1 and I2) did not exhibit increases in fitness. *indicates a significant difference between mean values of lines evolved with live vs heat-killed *S*. *marcescens*

The increased outcrossing rates within populations harboring variation could be due to outcrossing facilitating more rapid adaptation to *S*. *marcescens* than self-fertilization. After eight generations, only the lines harboring variation (M1,M2, and N) exhibited an increase in mean fitness in the presence of the parasite, relative to control populations evolved against heat-killed *S*. *marcescens* (Fig 3 and Table 2; M1, F_1,38_ = 9.07, P = 0.005; M2, F_1,38_ = 24.64, P < 0.001; N, F_1,38_ = 14.18, P < 0.001) Conversely, the inbred lines (I1 and I2) exposed to live parasites exhibited no significant change in fitness relative to their respective controls (Fig 3 and Table 2; I1, F_1,38_ = 0.116, P = 0.735; I2, F_1,38_ = 0.794, P = 0.378). Therefore, the populations that harbored variation and exhibited elevated rates of outcrossing adapted to *S*. *marcescens*, whereas the populations without variation evolved reduced rates of outcrossing and did not adapt to the parasite.

**Table 2:** ANOVA table for fitness assay.

Source	Sum of Squares	Df	Mean Square	F	P
Model	5.21	9	0.579	14.91	< 0.001
Error	1.47	38	0.038		
Total	6.68	47			
Treatment	1.00	1	1.00	25.96	< 0.001
Line	3.51	4	0.878	22.65	< 0.001
Treat X Line	0.985	4	0.246	6.17	< 0.01

## Discussion

Additive genetic variation is critical to outcrossing’s predicted selective advantages over self-fertilization. Outcrossing is predicted to facilitate rapid adaptation by uniting multiple beneficial alleles into a common genome, a process that requires unique beneficial alleles in different lineages [26, 37, 38]. Outcrossing may also prevent inbreeding depression by masking the expression of recessive deleterious mutations, which requires the presence of unique dominant alleles in different lineages [30, 31, 45]. Here, we found that, as predicted, variation is critical for the evolution of increased outcrossing under novel environmental conditions. When exposed to a novel parasite, elevated rates of outcrossing were favored in populations harboring genetic variation (Fig 1), whereas reduced rates of outcrossing evolved in inbred populations. We then tested both the degree of initial inbreeding depression and the degree of adaptation to the parasite to assess the benefits of outcrossing in genetically variable populations within our experiment. We detected initial inbreeding depression in only a subset of the populations harboring variation. However, we detected significant levels of adaptation to the parasite in all of the host populations that harbored variation, whereas we did not detect adaptive change in any of the inbred populations. Thus, the primary benefit conferred by outcrossing was rapid adaptation to the novel environment (Fig 3), but this benefit was only manifested in the lines that harbored initial genetic variation.

Because no significant inbreeding depression was detected in both the N and M1 lines (Fig 2), reductions in fitness due to expression of recessive deleterious mutations can be ruled out as the primary factor selecting for elevated outcrossing rates in nematodes exposed to live parasite. Although the M2 line exhibited inbreeding depression prior to selection (Fig 2), it is also unlikely that inbreeding depression was solely responsible for elevated outcrossing rates in populations from the M2 line. Populations within the M2 line also adapted to parasite (Fig 3), meaning elevated rates of outcrossing in the M2 line were not solely driven by inbreeding depression. Further, we did not observe a parasite treatment by mating type effect (Table 2), indicating that strong selection imposed by the parasites did not alter patterns of inbreeding depression relative to exposure to heat-killed parasites. Therefore, the increased fitness that accompanied elevated rates of outcrossing was primarily the result of adaptation to the parasite, rather than recovery from initial inbreeding depression. This indicates that outcrossing in lines with initial variation was due to the capacity of outcrossing to assemble beneficial combinations of alleles to facilitate adaptation. Although populations with some degree of inbreeding depression evolved greater rates of outcrossing in a previous study [20], the results of our study explicitly demonstrate that the value of outcrossing can go beyond just the ability to remedy fitness loss due to the expression of homozygous deleterious mutations.

As expected, the inbred lines exhibited reduced rates of outcrossing, relative to the lines with variation (Fig 1), and did not adapt to the novel environment (Fig 3). The overall lower outcrossing rates within the inbred populations may be due to these populations incurring the inherent cost of males while receiving no benefit in return from outcrossing. The lack of adaptation to the parasite is presumably due to the absence of standing variation upon which selection could act. However, unexpectedly, the inbred lines exposed to the parasite evolved significantly reduced rates of outcrossing relative to the inbred lines exposed to the heat-killed control. This is surprising because genetically homogeneous populations responded differently to the two different environments. One potential explanation for this difference could be a sex-specific effect of the parasite. *C*. *elegans* males facilitate outcrossing, so greater rates of male mortality could reduce outcrossing rates, as observed in the *C*. *elegans-B*. *thuringiensis* system [22]. Here, with no benefit to outcrossing in the inbred populations, sex-specific effects of the parasite may have effectively driven males out of the populations. Interestingly, no sex-specific effects of *S*. *marcescens* have previously been identified in this system. However, previous experiments have only utilized host populations harboring variation.

Genetic exchange between lineages provides no adaptive benefit without standing additive genetic variation [25, 27], yet still incurs the costs associated with outcrossing [2, 3]. This suggests that a population must have an adequate level of genetic variation, or a sufficient capacity to generate variation via mutation, above a certain threshold in order for outcrossing to be beneficial. Such a threshold could obstruct the evolution and maintenance of outcrossing in some populations, and may contribute to high levels of self-fertilization in some taxa [30, 45, 46]. This work reinforces the idea that sufficiently high levels of variation are of paramount importance to a population’s ability to adapt and ultimately survive in the face of novel ecological pressures. Indeed, species in the Solanaceae family that rely heavily on self-fertilization exhibit greater extinction rates than those that regularly outcross [47].

These results paired with data on patterns of selection for outcrossing in field work [9–13] make a strong case for the power of novel ecological conditions to favor outcrossing lineages. Furthermore, our data presented herein suggest that this holds true especially when genetic variation is present in host populations, meaning the population genetic consequences of outcrossing allowing for genetic exchange between lineages is key to the ability of outcrossing to facilitate adaptation. And as the greater potential to facilitate rapid adaptation has been demonstrated to be a powerful enough force to select for outcrossing under novel environmental conditions, our results further show that genetic exchange between lineages is of integral importance to evolution and maintenance of outcrossing.

## Supporting Information

S1 File Male frequency data. Raw counts of male and hermaphrodite individuals along a transect in experimental populations during experimental evolution.
(XLSX)

S2 File Fitness data. Raw competitive fitness data from experimental populations.
(XLS)

S3 File Inbreeding data. Raw competitive fitness data from outbred and inbreed ancestral populations.
(XLSX)
